# A Text-Book of Pharmacology, Therapeutics and Materia Medica

**Published:** 1886-01

**Authors:** 


**A Text-Book of Pharmacology, Therapeutics and Materia Medica.** By T. LANDER BRUNTON, M. D., D. Sc., F. R. S., Assistant Physician and Lecturer on Materia Medica, at St. Bartholomew's Hospital, London, etc., etc. Adapted to the U. S. Pharmacopœia, by F. H. Williams, M. D., Boston, Mass. Philadelphia: Lea Brothers & Co. 1885.

This work we welcome as a really valuable contribution to medical science. It is not a mere compilation of dry facts and technical details, but is a scientific treatise upon the subject, which will prove a mine of wealth both to students and practitioners. The author has been long known as one of the most diligent in the study of the physiological and therapeutical action of drugs. In the preface, he states that more than fifteen years ago he had a work on materia medica completely written and arrangements completed for its immediate publication. A desire to make some improvements and to establish certain statements by experiments, led him to delay its publication. As he went on, the labor grew; increased experience as a teacher led him to see that the arrangement of the original work could be improved upon, and the result was the re-writing of the whole volume. We would commend the example to the imitation of some of our American authors, believing that a few years' delay in publication of some books would (with persistent labor on the part of the authors) have resulted in a great increase of honor to the writers and satisfaction to the readers.

Section I., which is devoted to general pharmacology and therapeutics, contains the best treatise on the subject with which we are familiar. It deserves unqualified praise, and ought to be read by every practitioner. That portion of the book devoted to general pharmacy is very good, but the same information is as well, or better, given in some other books. As a whole, however, we confidently commend the book to our readers as one they ought to have. As is usual with the Leas, the "make-up" of the book is in every respect all that could be desired.

